# Associations Between Acute Conflict and Maternal Care Usage in Egypt: An Uncontrolled Before-and-After Study Using Demographic and Health Survey Data

**DOI:** 10.15171/ijhpm.2018.107

**Published:** 2018-11-21

**Authors:** Saji S. Gopalan, Richard J. Silverwood, Omar Salman, Natasha Howard

**Affiliations:** ^1^Department of Global Health and Development, Faculty of Public Health and Policy, London School of Hygiene & Tropical Medicine, London, UK.; ^2^Department of Medical Statistics, Faculty of Epidemiology and Population Health, London School of Hygiene & Tropical Medicine, London, UK.

**Keywords:** Acute Conflict, Maternal Care, Multi-Level Modelling, Egypt

## Abstract

**Background:** United Nations’ (UN) data indicate that conflict-affected low- and middle-income countries (LMICs) contribute considerably to global maternal deaths. Maternal care usage patterns during conflict have not been rigorously quantitatively examined for policy insights. This study analysed associations between acute conflict and maternal services usage and quality in Egypt using reliable secondary data (as conflict-affected settings generally lack reliable primary data).

**Methods:** An uncontrolled before-and-after study used data from the 2014 Egypt Demographic and Health Survey (EDHS). The ‘pre-conflict sample’ included births occurring from January 2009 to January 2011. The ‘peri-conflict sample’ included births from February 2011 to December 2012. The hierarchical nature of demographic and household survey (DHS) data was addressed using multi-level modelling (MLM).

**Results:** In total, 2569 pre-conflict and 4641 peri-conflict births were reported. After adjusting for socioeconomic variables, conflict did not significantly affect antenatal service usage. Compared to the pre-conflict period, periconflict births had slightly lower odds of delivery in public institutions (odds ratio [OR]: 0.987; 95% CI: 0.975-0.998; P<.05), institutional postnatal care (OR: 0.995; 95% CI: 0.98-1.00; P=.05), and at least 24 hours post-delivery stay (OR: 0.921; 95% CI: 0.906-0.935; P<.01). Peri-conflict births had relatively higher odds of doctor-assisted deliveries (OR: 1.021; 95% CI: 1.004-1.035; P<.05), institutional deliveries (OR: 1.022; 95% CI: 1.00-1.04; P<.05), private institutional deliveries (OR: 1.035; 95% CI: 1.017-1.05; P<.001), and doctor-assisted postnatal care (OR: 1.015; 95% CI: 1.003-1.027; P<.05). Sensitivity analysis did not change results significantly.

**Conclusion:** Maternal care showed limited associations with the acute conflict, generally reflecting pre-conflict usage patterns. Further qualitative and quantitative research could identify the effects of larger conflicts on maternal careseeking and usage, and inform approaches to building health system resilience.

## Background

Conflicts affect population health and human development adversely.^1^ Currently, 800 million people live in conflict-affected settings globally.^2^ In 2014, 40 active armed-conflicts in 46 countries caused 167 000 fatalities.^2,3^ Conflict is considered a major barrier to achieving United Nations (UN) Sustainable Development Goals (SDGs), including improved maternal health status.^4,5^ Conflicts in low- and middle-income countries (LMICs) are associated with poor maternal and child health (MCH) outcomes.^6^ For instance, among the 34 LMICs farthest from reaching global MCH targets, 22 are conflict-affected. Conflict-affected settings are considered to contribute a significant share of global maternal and child deaths (about 30% to 50%), though this is a source of ongoing debate.^6,7^

Inadequate maternal care-seeking is considered as a leading cause of poor MCH indicators in conflict-affected settings.^8^ During conflict, maternal care requires additional attention to emergency obstetric and newborn care along with routine antenatal (ANC) and postnatal (PNC) care visits.^6^ However, conflict-affected countries generally have less than half of the recommended numbers of health-workers or infrastructure necessary to address maternal care.^9-11^ Maternal and child deaths surge during and after conflict, mainly due to physical violence and the breakdown of healthcare delivery systems.^10^ Epidemiological estimates indicate that access to skilled birth attendants and ANC can reduce maternal deaths considerably (eg, by 33% and 12% respectively in LMICs), including during conflict.^6,12,13^ Although appropriate maternal care usage has been identified as the most effective means of improving MCH in conflict-affected settings, evidence on maternal care-seeking and usage patterns remains limited.^9^ Without better evidence on these patterns during conflict, policy and practice responses may not be effective or efficient.^6^

The Middle-East region historically known for persistent conflicts and Egypt is a conflict-prone country in the region.^3,14^ During 2011-2013, Egypt experienced acute conflict as thousands of civilians protested against the government.^15^ Prolonged armed and unarmed protests across the country disrupted governance and civil life. In addition to increased fatalities and injuries, the conflict was expected to weaken economic growth and human development indicators.^15^ Political paralysis damaged public service delivery systems, access to health services, and healthcare-seeking.^16^ The adverse effects of the conflict on health sector continued as there were prolonged health worker strikes as a result of the conflict, leading to disruption of healthcare services.^17^

Due to the inadequacy of existing data, few studies focus on maternal care usage in conflict-affected settings.^6^ While primary data are preferable when undertaking such uncontrolled before and after analyses, collection of primary data is often challenging during conflict.^1,18^ Reliable secondary data, especially a country-wide demographic and household survey (DHS), may be a feasible alternative option. Egypt was chosen for the following 3 reasons: (1) a recent conflict is better for investigating the effects of conflict on maternal care usage and provides fresh evidence, as the nature and trajectory of conflicts change over time; (2) the 2014 Egypt DHS provides country-wide data on comprehensive maternal care, enabling a before-and-after comparison of the effect of conflict; and (3) analysis of the effect of the Egyptian conflict on the health system and maternal care remains limited.^19^

## Objectives

The aim of this uncontrolled before-and-after study was to examine the association between the acute 2011-2012 Egyptian conflict and maternal care usage and quality, using 2014 Egypt DHS data. Objectives were to estimate: (*i*) the association of conflict with usage of antenatal, delivery, and postnatal services; and (*ii*) the association of conflict with quality of antenatal services received.

## Methods

### Study Setting

The pluralistic Egyptian healthcare system demonstrates inequities, with rural areas possessing poorer infrastructure, funding, and human resources than urban areas.^15,20^ Private out-of-pocket spending was 72% of total health spending in 2014, while total health spending was 5% of gross domestic product in 2011.^21^ The public sector, despite being the largest healthcare provider, faces constraints such as limited funding, staff, and managerial capacity.^16,20,22^ During the last decade, MCH scenario in Egypt has been showing some progress with growing inequalities.^21^ In 2012, the under-five mortality rate was 27 per 1000 live births while the maternal mortality ratio was 82 per 100 000 live births.^19^ Rural children were more at risk of anaemia than urban children (29% and 23% respectively) in 2014.^23^ A hospital-based study in 2009 indicated obstetric haemorrhage, hypertensive disorders of pregnancy and cardiac arrest were major causes of maternal deaths.^24^ Poor quality care and delay in seeking care were also other reasons reported for maternal deaths.^24^

The Egyptian revolution began in January 2011, and its acute phase continued until 2013.^15^ Media reports indicate the country is still at risk of conflicts among different political groups.^14^ The acute phase began when thousands of civilians protested against the Government, led by then long-time president Hosni Mubarak.^14,15^ Despite his resignation, conflicts continued and became widespread under the military regime. When an elected government took office in 2012, protests became more acute.^15^ Contributing factors to the rise of mass protest, included an autocratic government, rising poverty, and inequitable social programmes.^14,15^ Major events related to the revolution is described in Figure.

**Figure:**
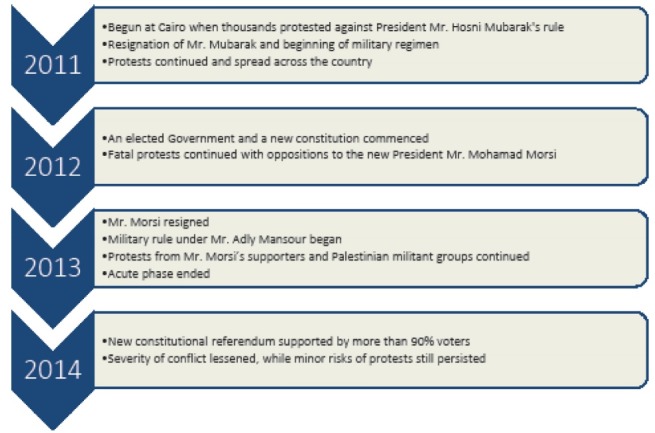
Major Events Related to Egyptian Conflict 2011.
Source: Adapted from Abdou DS^15^ and Maher S.^14^

### Study Design

A quasi-experimental ‘uncontrolled before-and-after’ design was selected to explore the relationship between conflict and maternal care usage. Uncontrolled before-and-after studies enable assessment of the relationship between 2 events or interventions, when a typical control is not present but a pre-post comparison is possible.^25^ This design enabled comparison of changes in levels of maternal care usage and quality of care before and during acute conflict. Multi-level modelling (MLM) was applied as it enabled exploration of an association between 2 phenomena within DHS data (as DHS analysis units are hierarchical),^26^ accounted for the clustered nature of the data, and reduced the chance of type 1 error.^27,28^ Births occurring from January 1, 2009 to January 31, 2011 constituted the ‘pre-conflict’ sample and those from February 1, 2011 to December 31, 2012 were the ‘peri-conflict’ sample. While even acute conflicts are rarely ‘fixed-period shocks’ the way natural disasters can be, authors selected this conflict period in Egypt as multiple data sources indicated it was particularly acute, meaning it could be modelled as a fixed period to examine the effects of conflict on service usage.

The study adopted a working definition of ‘conflict-affected’ from the relevant literature^1-3,6,10,18^ as a setting in which routine socio-political, economic and/or civil life are disrupted due to armed political conflict. Births occurring from January 1, 2013 onwards were excluded, as media reports indicated that acute conflict ended in early 2013.

### Data Source and Sampling

Data were drawn from the Egypt Demographic and Household Survey (EDHS) 2014,^19^ a nationally representative study providing both national and sub-divisional data, though excluding North and South Sinai governorates for political reasons. Authors examined other datasets, eg, ACLED that uses media reports to estimate the spatial distribution of violence, and earlier DHS datasets (eg, 2008 and 2005) to compare violence distribution during conflict and maternal care levels prior to the ‘Arab Spring.’ However, the 2014 EDHS was determined as most appropriate for robust before-and-after analysis, as other sources did not fit the type of analysis.

EDHS data were collected in April-June 2014.^19^ The EDHS 2014 used multi-stage sampling, with towns and villages as primary sampling units (PSU) for urban and rural areas respectively.^19^ First, 884 PSUs were selected. Second, depending on PSU size, systematic sampling yielded 1-3 parts per PSU (1000 households each). Third, each part was divided into equally-sized segments (200 households each), 2 to 3 of which were selected randomly from each PSU. A total of 1838 segments (clusters) were selected from 884 PSUs. A household listing was undertaken in each segment. An average of 15 households was selected from each segment using a systematic random sample procedure. Thus, yielding a total of 29 471 households for the 2014 EDHS. Eligible participants were ever-married women aged 15-49 and present in selected households the night prior to interview. There were a total of 21 903 eligible women for the entire survey. From each woman, details on childbirths for the preceding five years from the date of survey were gathered.

Between January 1, 2009 and December 31, 2012, there were 7210 births recorded from 7118 eligible women in 1679 clusters as per EDHS. These 7210 births came from both pre-conflict and peri-conflict periods. Specifically, the pre-conflict period had 2569 births and the peri-conflict period had 4641 births.

### Data Collection and Outcome Measures

Local enumerators, who were recent university graduates, collected data using an Arabic version of the DHS questionnaire. All questions were pre-tested and revised to suit the local context. This study used the component survey of ever-married women aged 15-49 years, focusing on usage of MCH services. The questionnaire collected data on respondent background, reproduction, pregnancy and breastfeeding, child immunisation and health, husband’s background, and respondent employment and decision-making. Maternal care data included location of care-seeking (home, institution), facility type (public, private), provider type (skilled, unskilled), and frequency, timeliness, and content of services received.

Study outcomes were components of maternal care usage, as maternal healthcare is usually categorised by maternity stage, ie, antenatal, delivery, and postnatal (Table 1). Each outcome was analysed by contextual variants, ie, location (facility, non-facility), provider (skilled, unskilled), and number of ANC or PNC visits. Explanatory variables were mother’s age (<25, 25-29, 30-34, >35), education (no education, primary, secondary and above), occupation (currently working and not working), birth order (1, 2-3, 4-5, 6 and above), child gender, residence (urban/rural), and household wealth quintile.

**Table 1:** Outcome Measures

**Maternal Care**	**Variables**
ANC	Whether ANC received
	At least 4 ANC received
	Whether ANC received from a public institution
	Whether ANC received from a skilled provider
	Whether ANC received from a doctor
	Whether first ANC received within 12 weeks*
ANC* content	Whether ANC included blood pressure measurement
	Whether ANC included weighing
	Whether ANC included collection of urine samples
	Whether ANC included collection of blood samples
	Whether ANC included prescription of iron tablets
Adequate ANC quality*	Whether ANC included all recommended elements, ie, blood pressure, weight, urine and blood samples, iron tablets
Delivery care	Whether delivery occurred in an institution
	Whether delivery occurred in a public institution
	Whether delivery occurred in a private institution
	Whether delivery assisted by a skilled provider
	Whether delivery assisted by a doctor
	Post-delivery stay at least 24 hours
PNC*	Whether PNC received
	Whether PNC received from a skilled provider
	Whether PNC received from an institution
	Whether PNC received from a public institution
	PNC received within 24 hours
	PNC received within 48 hours

Abbreviations: ANC, antenatal care; PNC, postnatal care. * Only available for most recent birth.

Wealth quintiles were derived from the EDHS wealth index, which weighted household characteristics and asset possession using principal component analysis (PCA) of their relative importance.^19^ The index categorised sampled households into quintiles from poorest to wealthiest. ANC quality was considered adequate if the woman reported receipt of all five key services: (*i*) being weighed, (*ii*) having blood pressure measured, (*iii*) having a urine sample collected, (*iv*) having a blood sample collected, and (*v*) having iron tablets prescribed. This variable was binary, taking value 1 if all services were received and 0 if one or more of these services were not received.

### Data Analysis

Data analysis, performed using Stata version 13, included (*i*) descriptive analyses; (*ii*) estimates of effects through MLM; and (*iii*) sensitivity checks. *Descriptive analyses* were undertaken on key explanatory variables. Variables were summarised by period (pre-conflict, peri-conflict), using means for continuous variables and frequencies for categorical variables.

*MLM analysis* enabled investigations of variance within and between clusters,^29^ as EDHS data are hierarchical (ie, births nested within households, households within clusters, clusters within PSUs, PSUs within Governorates).^19^ Conventional regression models do not account for data hierarchy and may underestimate standard errors of the effect sizes, increasing the likelihood of type 1 error.^25,29-31^ Multilevel regression models account for data clustering and correct for the dependency of observations within a cluster (eg, primarily multiple women per household, though multiple births per woman were also accounted for).^28,32^ While a clustered standard errors approach can be more practical, advantages of MLM include that it provides separate estimates for individual states and can address unbalanced data (eg, differing sample sizes in different states). Similarly, fixed effects models can offer improvements for causal estimation by removing potential unobserved confounding at higher levels from parameter estimation, but this also reduces potentially relevant variation. Thus, an MLM or ‘random effects’ model was selected.

In MLM, the fixed component is a linear function of individual and contextual factors, while the random component represents variance between units within the same level.^30,31^ This analysis considered the sample hierarchy at four levels: births (level 1), nested within clusters (level 2), nested within PSUs (level 3), and nested within governorates (level 4). The study considered women at the same level of analysis as births, since few women had multiple births.^19^

Multilevel logistic regressions were performed adjusting for mother’s age, education, residence, employment status, household wealth, child gender, and birth order. Since the DHS applies sampling weights to the sample for national representativeness, these weights were applied in the multilevel regressions.^19^ Sampling weights were rescaled, since including raw weights without scaling in an MLM leads to biased parameters and standard errors.^31,33^ In this analysis, weights were scaled so that the new weights summed to the effective cluster size.^34^

The following model specification was used:

$$\begin{array}{l} \log\left(\frac{Y_{ijkl}}{1-Y_{ijkl}}\right)=\\ \alpha +\beta_{1}X_{1ijkl}+...+\beta_{n}X_{nijkl}+\gamma T_{ijkl}+\\ \theta_{1}+\mu_{kl}+\eta_{jkl}+\varepsilon_{ijkl} \end{array}$$

Where Y_ijkl_ is the binary outcome for birth i (level 1) within cluster j (level 2) within PSU k (level 3) within governorate l (level 4). α is a constant; X_1ijkl ……._ X_nijkl_ are the aforementioned covariates with β_1 ……_ βn as their coefficients; T_ijkl_ is a binary variable coded 1 for the peri-conflict period and 0 for the pre-conflict period, with γ as its coefficient; θ_l_, µ_kl_, η_jkl_, and ε_ijkl_ are the error terms at governorate, PSU, cluster, and birth levels respectively.

*Sensitivity analysis* assessed the effects of alterations of study definitions and model specifications in 4 ways. First, varying the cut-off for onset of conflict (January 2011 versus February 2011 versus March 2011). Second, changing levels of analysis (4-level versus 3-level model). Third, applying sampling weights (no weights versus weighted unscaled versus weighted rescaled). Fourth, dropping births that took place closer to conflict onset.

## Results

### Sample Characteristics

Table 2 shows the distributions of socioeconomic and demographic variables for 2569 births in the pre-conflict period and 4641 in the peri-conflict period. This unexpected near doubling of births in the peri-conflict period compared to the pre-conflict period was described by the DHS report as due to an unusual national doubling of births from 2011 to early 2014 (eg, total numbers of stillbirths to women aged 15-49 averaged 9783 during 2011-2012 and only 4055 during 2009-2010^19^). There was a marked shift to younger maternal age between pre-conflict and peri-conflict periods. In the pre-conflict period 66% of births were to women aged 30+ years, but by the peri-conflict period this had declined to 41%. Most births were to women educated to secondary level or above (58.2% pre-conflict, 61.7% peri-conflict), living in rural settings (65.1% pre-conflict, 69.2% peri-conflict). Slightly above half (55% pre-conflict and 52.9% peri-conflict) of births were boys and most mothers already had 2-3 children (60.1% pre-conflict, 54% peri-conflict). No notable differences were found in wealth status between conflict periods, though most births (23.8% pre-conflict, 25.1% peri-conflict) were to women in the middle quintile and non-working women (84.9% and 86.7% respectively). In both periods, approximately 97% of births were to Muslim women, while the rest were Christian.

**Table 2:** Sample Characteristics for Births

**Characteristics**	**Pre-conflict** **n = 2569**		**Peri-conflict** **n = 4641**	
	**No.**	**%**	**No.**	**%**
Age group				
<25	157	6.1	1040	22.4
25-29	709	27.6	1713	36.9
30-34	853	33.2	1091	23.5
>35	850	33.1	798	17.2
Education				
No education	550	21.4	840	18.1
Primary	524	20.4	937	20.2
Secondary and above	1495	58.2	2863	61.7
Residence				
Urban	897	34.9	1429	30.8
Rural	1672	65.1	3212	69.2
Gender of child				
Male	1413	55	2455	52.9
Female	1156	45	2186	47.1
Wealth index				
Poorest	465	18.1	784	16.9
Poorer	504	19.6	928	20.0
Middle	611	23.8	1165	25.1
Richer	516	20.1	993	21.4
Richest	473	18.4	770	16.6
Currently working				
No	2181	84.9	4024	86.7
Yes	388	15.1	617	13.3
Birth order				
1	252	9.8	1109	23.9
2-3	1544	60.1	2506	54.0
4-5	622	24.2	863	18.6
6 and above	154	6.0	162	3.5
Religion				
Muslim	2479	96.5	4488	96.7
Christian	90	3.5	158	3.4

### Effect of Conflict on Antenatal Service Usage and Quality 

Table 3 shows both unadjusted and adjusted estimates of the association between conflict and antenatal service usage. After adjusting for socioeconomic (ie, age, education, residence, wealth, working status) and biological determinants (ie, child gender, birth order), no associations between conflict and ANC usage were found.

**Table 3:** Multilevel Modelling Estimates of the Association of Conflict With ANC Usage and Quality

	**Means**		** OR (95% CI)**	
	**Pre-conflict**	**Peri-conflict**	**Unadjusted**	**Adjusted** ^a^
Any ANC visit	88.3	90.4	1.029** (1.008-1.050)	1.014 (0.995-1.033)
Had 4 or more ANC visits	92.0	92.8	1.012** (1.003-1.020)	1.008 (0.996-1.019)
ANC received from a government provider	17.4	16.4	0.987 (0.971-1.030)	0.987 (0.973-1.001)
ANC received from a doctor	61.5	62.1	1.005 (0.978-1.033)	1.006 (0.980-1.032)
First ANC received within 12 weeks	26.0	56.6	1.030 (0.990-1.060)	1.020 (0.990-1.040)
Weighed during ANC	90.1	89.4	0.999 (0.981-1.017)	1.000 (0.977-1.024)
Blood pressure measured during ANC	93.2	93.9	1.009*** (1.004-1.014)	1.007 (0.997-1.018)
Urine sample given during ANC	75.7	77.2	1.021* (1.002-1.041)	1.005 (0.975-1.036)
Blood sample given during ANC	77.2	79.8	1.031*** (1.019-1.043)	1.020 (0.997-1.042)
Iron tablet received during ANC	64.0	66.6	1.039*** (1.016-1.062)	1.015 (0.987-1.044)
Adequate ANC quality	49.7	53.2	1.035*** (1.012-1.057)	1.022 (0.996-1.048)

Abbreviations: OR, odds ratio; ANC, antenatal care. ^a^ Multilevel modelling estimates adjusted for age, education, residence, wealth, working status, child gender and birth order; * <.05; ** <.01; *** <.001; sample size – pre-conflict 2569; peri-conflict 4641.

### Effect of Conflict on Delivery Service Usage

Table 4 shows that the adjusted odds of doctor-assisted deliveries were 2% higher (odds ratio [OR]: 1.021; 95% CI: 1.004-1.035; *P* < .05); odds of institutional delivery were 2% higher (OR: 1.022; 95% CI: 1.004-1.039; *P* < .05); odds of delivering in a private institution were 3% higher (OR: 1.035; 95% CI: 1.017-1.053; *P* < .001); odds of delivering in a public institution were 1% lower (OR: 0.987; 95% CI: 0.975-0.998; *P* < .05); and odds of a woman’s post-delivery stay lasting for at least 24 hours were 8% lower (OR: 0.921; 95% CI: 0.906-0.935; *P* < .01) for peri-conflict births compared to pre-conflict births.

**Table 4:** Multilevel Modelling Estimates of Association of Conflict With Delivery Service Usage

	**Means**		**OR (95% CI)**	
	**Pre-conflict**	**Peri-conflict**	**Unadjusted**	**Adjusted** ^a^
Delivery by skilled provider	91.3	92.5	1.017* (1.001-1.034)	1.012 (0.999-1.024)
Delivery by doctor	87.8	89.8	1.027** (1.008-1.045)	1.021* (1.004-1.035)
Delivery in an institution	86.2	88.4	1.029** (1.009-1.05)	1.022* (1.004-1.039)
Delivery in a public institution	28.2	26.1	0.977*** (0.966-0.988)	0.987* (0.975-0.998)
Delivery in a private institution	57.5	61.6	1.053*** (1.037-1.07)	1.035*** (1.017-1.053)
Post-delivery stay at least 24 hours	58.3	50.7	0.929** (0.911-0.948)	0.921** (0.906-0.935)

Abbreviation: OR, odds ratio. ^a^ Multilevel modelling estimates adjusted for age, education, residence, wealth, working status, child gender and birth order; * <.05; ** <.01; *** <.001; sample size – pre-conflict 2569; peri-conflict 4641.

### Effect of Conflict on Postnatal Service Usage

Table 5 shows that the adjusted odds of doctor-assisted PNC were 2% higher (OR: 1.015; 95% CI: 1.003-1.027; *P* < .05) and the odds of receiving PNC from an institution were 1% lower (OR: 0.9; 95% CI: 0.98-1.00; *P* = .05) for peri-conflict versus pre-conflict births.

**Table 5:** Multilevel Modelling Estimates of the Association of Conflict With PNC Service Usage

	**Means**		**OR (95% CI)**	
	**Pre-conflict**	**Peri-conflict**	**Unadjusted**	**Adjusted** ^#^
Had any PNC	32.0	33.8	1.022* (1.005-1.039)	1.008 (0.987-1.030)
PNC received within 24 hours	24.5	24.7	1.00 (0.969-1.031)	1.005 (0.979-1.032)
PNC received within 48 hours	37.7	35.9	0.977 (0.922-1.036)	0.983 (0.928-1.041)
PNC received from a skilled provider	99.9	99.6	0.998 (0.993-1.003)	0.999 (0.995-1.003)
PNC received from a doctor	96.0	96.8	1.008 (0.994-1.022)	1.015* (1.003-1.027)
PNC received in an institution	97.5	97.6	0.999 (0.993-1.005)	0.995* (0.980-1.000)
PNC received in a public institution	17.7	16.6	0.987 (0.956-1.02)	0.996 (0.963-1.030)
PNC received in a private institution	81.7	83.0	1.015 (0.977-1.054)	1.009 (0.971-1.049)

Abbreviations: OR, odds ratio; PNC, postnatal care. ^a^ Multilevel modelling estimates adjusted for age, education, residence, wealth, working status, child gender and birth order; * <.05; ** <.01; *** <.001; sample size – pre-conflict 2569; peri-conflict 4641.

### Sensitivity Analysis

Sensitivity analyses showed that including alternative study definitions and model specifications did not yield different results from those in the main analysis, except slight variations for a few variables (Supplementary file 1, Table S1). A goodness-of-fit analysis, not shown, demonstrated that models with 4 analysis levels provided a better fit than 3-level models.

## Discussion

### Primary Findings

This study is one of the first attempts to use DHS data to quantify the association between conflict and maternal services usage. Given the general lack and low quality of primary data in conflict-affected settings, DHS is perhaps the most reliable current data source to explore associations between conflict and maternal health.^19^ This study identified minor associations, both negative and positive, between the acute 2011-2012 Egyptian conflict and usage of selected maternal services. Overall, maternal services usage during the conflict was not found to be noticeably different from routine usage in Egypt. However, MLM appeared to work well and similar modelling approaches could be applied in more severe conflicts or to examine healthcare usage for other reproductive health issues. As the existing literature assessing the impact of conflict on maternal care is limited, authors compared findings with other studies reporting maternal care levels in conflict-affected settings, despite these generally not being able to attribute any observed changes to conflict. Due to the scarcity of evidence, comparisons were expanded beyond acute to include chronic conflicts.

This study was a unique attempt to assess the association of conflict with post-delivery stay. As optimum post-delivery stay is a pre-requisite to reducing postpartum deaths, its inclusion in maternal care assessment is important. However, its assessment is not common and these findings may inspire researchers to examine delivery stay in maternal care analyses, especially in conflict-affected settings. Among the negative impacts of conflict was an estimated 8% lower odds for staying at least 24 hours following delivery during conflict. The average maternity ward stay during conflict was 16 hours (not shown in results), considerably less than the World Health Organization (WHO) recommended minimum of 24 hours for uncomplicated vaginal delivery.^35^ This finding is important as reduced facility stay is known to increase morbidity and mortality risks to both mothers and newborns.^35,36^ Epidemiological evidence indicates that almost 50% of maternal deaths globally, and 40% in Egypt, occur within 24 hours after birth.^35,37,38^ The literature postulates that the threat to life of patients and health professionals may shorten delivery stays during acute conflict.^39-42^ In some places, hospitals are at risk of violence and mothers prefer to leave with their babies immediately after delivery.^43,44^ However, we found no literature indicating that the Egyptian conflict threatened the lives of patients and professionals resulting in shorter postpartum stays. Thus, further research is needed to explore possible mediators of the shorter average stay noted.

Findings indicated a slight decline in use of public institutions for delivery during conflict. Relatively lower dependence on public institutions for childbirth was a noticeable recent trend in Egypt,^38^ and the literature is mixed on facility choice for delivery during acute conflicts.^45-48^ It may not be an issue of trust, but rather accessibility and safety when choosing a provider during conflict.^49^ In Egypt, women reported limited capacity in public institutions as a reason for choosing private providers.^38^ Even during conflict, they may have considered that private providers could ensure a safer delivery than their public-sector counterparts.

Findings indicated no association between conflict and ANC usage. The literature shows a mixed trend in ANC service usage during conflict.^49-51^ Provisional facility-based arrangements in Pakistan during acute conflict suggested higher rates of ANC usage and earlier initiation of ANC visits.^40^ Community outreach also enhanced early ANC usage during conflicts in South Asia and Africa.^40,49,51^ Alternatively, during the 2006 Lebanon conflict, women reported delayed initiation of ANC.^41^ A Nepal study reported a decline in early ANC use and numbers of ANC visits during conflict.^49^ Increased policy attention and initiatives to improve ANC quality in Egypt in the last decade have led to gradual improvement in reported adherence to ANC clinical guidelines.^38^ The 2% increased odds in adequate ANC quality during conflict could be due to this ongoing focus on enhancing ANC quality. As the conflict was not severe in violence or duration, providers would still have been expected to comply with treatment protocols. Other studies indicate that prevailing healthcare quality may not be compromised if conflicts are not severely life-threatening.^12,52^ In contrast, during severe armed conflicts in Afghanistan and Yemen, quality of maternal care worsened drastically,^53,54^ while quality of care in refugee camps was mixed depending on contextual factors.^44,52^

The slight increases in odds of institutional deliveries, doctor-assisted deliveries, and childbirth in private institutions support recent trends in Egypt seen in EDHS findings for 2009-2014.^19^ Even during acute conflict, Egyptian women preferred institutional deliveries, particularly in private institutions. Limited trust in public institutions, especially primary and community-level, was identified as a reason to choose private institutions for delivery.^16,21,55^ This preference would have increased the likelihood of doctor-assisted deliveries.^23^ The literature is mixed on delivery location during acute and mild conflicts. Due to poor-quality services, women chose home deliveries during the acute conflict in Lebanon.^41^ Similarly, a study including several sub-Saharan African countries reported reduced skilled birth attendance during conflicts.^56^ A study among Liberian women in Buduburam refugee camp in Ghana showed that reproductive health services were less prioritised, leading to reduced usage of essential maternal services.^57^ However, a Nepal study reported increased skilled birth-attendance and institutional deliveries during conflict.^45^

Findings indicated a slight conflict-associated increase in any PNC usage and doctor-assisted PNC, and a decline in institutional PNC. This decline is most likely related to early post-delivery discharge during conflict. Studies from other settings reported that women preferred to informally consult a community-based health-worker for postpartum health concerns.^40,58,59^ Without any perceived need or emergency, they were less likely to seek PNC.^58^ Low PNC usage is typical in conflict-affected settings, unless women access refugee camps.^6^ For example, only 20% use of any PNC was reported during conflict in Yemen,^60^ and only 36% reported in Palestine due to limited skilled personnel.^61^

### Implications

When analysing pathways of maternal care-seeking during conflict, 3 key drivers are availability of healthcare services, existing maternal care use patterns, and conflict severity.^62^ In the Egyptian context, a reason that larger adverse effects of conflict on maternal care or services usage were not found could be that the conflict was relatively minor and did not significantly challenge the health system.^15^ Secondly, the Egyptian healthcare system was relatively well-developed with reasonable levels of maternal care usage, compared to several other conflict-affected countries globally.^16^ Recent Egyptian health system reforms included countrywide initiatives to improve availability and use of maternal care among different population groups. These helped the country perform well against maternal health targets^15^ and could have contributed to increased resilience, helping retain similar levels of maternal care pre/peri-conflict. Experiences from Nepal similarly indicated that a resilient health system could meet many conflict-related challenges while protecting healthcare for mothers and children.^12^ However, health system resilience is a complex concept. While this interpretation of findings could encourage conflict-prone countries to strengthen health systems to increase resilience, further research is necessary to determine whether and how this may have contributed to findings. While it would be useful to use MLM methods to examine the effects of conflict on maternal services in a more significant conflict, pre/peri-conflict data of sufficient quality were not available at the time of this study to attempt this in countries affected by larger conflicts.

The association of the 2011-2012 Egyptian conflict with health service usage was not well documented. Media reports indicated the conflict did not intentionally disrupt healthcare delivery, though indirect disruptions were likely, and overall damage to governance and economy could have adversely affected both the health system and maternal service use.^15^ Thus, policy and practices designed to strengthen community-facility linkages could increase health system resilience to future shocks. For example, coordination of community-based networks with primary health facilities may help encourage women to access services in a timely manner and bring women to health centres safely.^63^ If mothers feel staying at health facilities post-delivery is risky during acute conflict, trained and supplied community-based networks could monitor the health of mothers and babies to reduce health risks. Community-level support systems and women’s groups have helped in meeting maternal health objectives in conflict-affected Myanmar, Pakistan, Sri Lanka, and the Philippines.^58,64-66^ Similarly, local non-governmental organizations (NGOs) in the Middle-East and Southeast Asia have motivated pregnant women to use maternity services.^41,67^

The Egyptian health system devolves authority locally.^20^ Local authorities need adequate autonomy to implement remedial measures addressing maternal care during emergencies.^12^ During other conflicts in the region, national governments have reportedly had limited scope and governance capacity to address maternal needs.^4^ Further, maternal health needs can be addressed more feasible locally than nationally during conflicts, as shown in Myanmar, Nepal, Pakistan, and Palestine.^40,41,49,58,68^

The main contribution of this study is its unique attempt to use secondary DHS data to quantify associations between conflict and maternal services usage using rigorous MLM methods, and showing that these can be applied to other research questions in similar contexts. Qualitative research is also essential to explore the contextual determinants of service usage and relevant remedial measures in Egypt. Research is also needed to understand how health systems in Egypt and other conflict-prone countries can minimise the risks to maternal health during conflict.

### Limitations

Several potential limitations should be noted, particularly related to secondary data analysis and DHS data specifically. First, primary research would have secured additional relevant data, beyond the scope of the DHS (eg, access to emergency obstetric care). Second, given the country-wide geographical spread of the conflict and lack of data on region-specific exposures, this study considered all women to be equally exposed to conflict and could not differentiate level of exposure to conflict. Third, although conflict continued for a longer period, a shorter study period was selected due to availability of reliable data for this period. Similarly, underlying temporal trends could have affected observed associations, although the period under consideration was too short for a large temporal trend to occur. Fourth, DHS data are susceptible to biases (eg, recall and social desirability) as mothers must recall details of their pregnancy and childbirth experiences over several years,^19^ and its sampling frame is limited to households and thus excludes homeless and institutionalised women.^19^ DHS did not cover North and South Sinai regions due to chronic political instability. Additionally, the unexpected differences in sample size between pre-conflict and peri-conflict periods indicate internal validity must be judged carefully, particularly given this study relied on DHS birth-rate data and assessing validity of DHS data collection and analysis was beyond study scope.^69^ Therefore, study findings can only be generalised to the areas included in analysis. Fifth, as effect sizes were generally small, interpretation of results should be cautious. Finally, qualitative data analysis could help corroborate the influence of contextual factors on maternal care usage. Despite these limitations, this is one of the first attempts to analyse the effect of conflict on maternal services use in Egypt or more generally.

## Conclusion

This study analysed DHS data to estimate the effects of conflict on maternal care usage. Overall, the acute conflict did not appear to have a major impact on maternal care usage as maternal care usage patterns during conflict were generally similar to recent trends in the country. This study did not find that conflict significantly affected ANC service use, while small positive associations were found in ANC quality score, institutional delivery, doctor-assisted childbirth, and private institutional attendance for delivery and PNC. This study found slightly reduced odds of post-delivery stay of at least 24 hours, childbirth at public institutions, and institutional PNC usage. Further studies are required to fully assess the effects of conflict on maternal morbidity and mortality, investigate contextual drivers of maternal care usage, and identify potential ways to improve health system resilience to support maternal needs in future conflicts.

## Ethical issues

Not applicable for secondary data analyses.

## Competing interests

Authors declare that they have no competing interests.

## Authors’ contributions

SSG, RJS, and NH conceptualised the study. SSG analysed data and drafted the first version. RJS and NH contributed to interpretation and critical review. All authors approved the version for submission.

## Authors’ affiliations

^1^Department of Global Health and Development, Faculty of Public Health and Policy, London School of Hygiene & Tropical Medicine, London, UK. ^2^Department of Medical Statistics, Faculty of Epidemiology and Population Health, London School of Hygiene & Tropical Medicine, London, UK.

## Supplementary files

Supplementary file 1 contains Table S1.

## Key messages

**Implications for policy makers**

- Associations between conflict and maternal care were limited and usage generally similar to pre-conflict patterns.
- Any maternal health strategy should consider potential effects of conflict and violence on services, including post-delivery stay, to mitigate disruption to maternal access and usage.
- Ensuring availability of health data in conflict-affected settings would allow quantitative modelling of the effects of larger conflicts on maternal care-seeking, usage, and quality.

**Implications for public**

This study demonstrates the applicability of multi-level modelling (MLM) analysis on hierarchical secondary data while exploring associations between conflict and maternal care. Results inspire further research into maternal care provision and usage in conflict-affected settings. Further research is needed on the role of contextual factors driving maternal service usage during conflicts and potential approaches to building health system resilience.
