# Supplementary material for: Associations Between Acute Conflict and Maternal Care Usage in Egypt: An Uncontrolled Before-and-After Study Using Demographic and Health Survey Data
Source: Int J Health Policy Manag. 2018 Nov 21;8(3):158–67. doi: 10.15171/ijhpm.2018.107 (PMC6462197; doi:10.15171/ijhpm.2018.107)
Supplement: Supplementary file 1 — contains Table S1. [file ijhpm-8-158-s001.pdf]

Supplementary file 1

Among the various sensitivity analyses conducted, Table S1 only presents the results which varied between the main model and the alternate models. For births that occurred closer to onset of conflict (ie, just before and just after), sensitivity of the outcomes showed the odds of receiving ANC from a government provider decreased during conflict than pre-conflict. There could be the possibility of misclassification of a particular maternal health event vis-à-vis exposure status to the conflict due to the continuum of care required for maternal health (ANC followed by delivery and PNC). For instance, a birth that occurred during the early conflict period may have had its ANC in the pre-conflict period. When the sample weight was changed only the main model showed a reduced odds of obtaining institutional PNC during conflict, while only the alternate model (ie, weighted unscaled) showed an increase in the odds of obtaining adequate ANC quality during quality. However, weighting of the sample was more valid in DHS data to balance for the differences in the sample size at various population strata. When changing the levels of analysis, only the main model showed a reduction in the odds of receiving institutional delivery and institutional PNC and a slight increase in the odds of obtaining doctor-assisted PNC.

Table S1. Sensitivity Analyses

**Changing the sample weighting**			
	**Unweighted OR (95% CI)**	**Weighted Unscaled OR (95% CI)**	**Weighted Rescaled (Main Analysis) OR (95% CI)**
Adequate ANC quality	1.022 (0.992-1.051)	1.032* (1.004-1.061)	1.022 (0.996-1.048)
PNC received in an institution	0.995 (0.981 - 1.009)	0.997 (0.984-1.010)	0.995* (0.980-1.000)
**Changing the levels of analysis**			
	**Three levels[$] OR (95% CI)**		**Four levels[#] (Main analysis) OR (95% CI)**
Delivery in a public institution	0.988 (0.964-1.012)		0.987* (0.975-0.998)
PNC received from a doctor	1.015 (0.996-1.034)		1.015* (1.003-1.027)
PNC received in an institution	0.995 (0.981-1.009)		0.995* (0.980-1.000)
**Changing the sample size for ANC**			
	**Omitted births from Jan 2011 to Sep 2011 OR (95% CI)**		**Full birth sample (Main analysis) OR (95% CI)**
ANC received from government provider	0.975** (0.957-0.993)		0.987 (0.973-1.001)

#Multilevel modelling estimates adjusted for age, education, residence, wealth index, currently working status, child gender and birth order; * <0.05; ** <0.01; *** <0.001; table presents only those variables where statistically significant results are different across models
